# Association of Chorioamnionitis with Early and Late Neonatal Sepsis in Preterm Infants with Gestational Age < 32 Weeks

**DOI:** 10.3390/diagnostics16081125

**Published:** 2026-04-09

**Authors:** Evgeniya Babacheva, Dimitrios Rallis, Marina Malakozi, Katerina Tzafilkou, Efthimia Papacharalampous, Ilias Chatziioannidis, Paraskevi Liouliou, Evangelia Giannousiou, Maria Florou, Maria Tzitiridou-Chatzopoulou, Christos Tsakalidis, Maria Lithoxopoulou

**Affiliations:** 1 2nd Department of Neonatology and NICU, Aristotle University of Thessaloniki, “Papageorgiou” Hospital, 56403 Thessaloniki, Greece; ebbabacheva3@gmail.com (E.B.); drallis@uoi.gr (D.R.); katerinatzaf@gmail.com (K.T.); efthpap@gmail.com (E.P.); vi_liou07@windowslive.com (P.L.); evi_giann@yahoo.com (E.G.); tsakalidisx@gmail.com (C.T.); 2 1st Department of Neonatology and NICU, Aristotle University of Thessaloniki, “Hippokratio” Hospital, 56642 Thessaloniki, Greece; malakozim@gmail.com (M.M.); ihatzi@auth.gr (I.C.); 3 2nd Department of Pediatric Surgery, Aristotle University of Thessaloniki, “Papageorgiou” Hospital, 56403 Thessaloniki, Greece; flwrou.mar@gmail.com; 4 Department of Midwifery, School of Healthcare Sciences, University of Western Macedonia, Keptse, 50200 Ptolemaida, Greece

**Keywords:** chorioamnionitis, prematurity, early onset sepsis, late onset sepsis

## Abstract

**Background**: Chorioamnionitis (CA) is a major pathological cause of preterm labor and is associated with both short- and long-term adverse outcomes in neonates, including early-onset sepsis (EOS) and late-onset sepsis (LOS). Neonatal sepsis remains a significant contributor to morbidity and mortality in neonatal intensive care units (NICUs). **Aim**: This study aimed to evaluate the association between maternal chorioamnionitis and the incidence of early-onset and late-onset neonatal sepsis in preterm neonates born at <32 weeks’ gestation. Furthermore, the study investigated maternal and neonatal factors affecting the presentation of sepsis. **Methods**: A retrospective cohort study was conducted on the medical records of preterm neonates born between 2020 and 2022. Inclusion criteria were gestational age < 32 weeks, available microbiological or histological examination for chorioamnionitis, and complete maternal medical records. Infants were categorized into two groups based on the presence (CA group) or absence (non-CA group) of histological and/or microbial chorioamnionitis. Descriptive statistical analyses were performed, including calculation of frequencies and percentages for categorical variables and means with standard deviations and ranges for continuous variables. **Results**: A total of 189 neonates were included, with a mean birth weight of 1286 ± 405 g and a mean gestational age of 29.2 ± 2.1 weeks. The CA group consisted of 55 neonates (29.1%), while 134 (70.9%) were in the non-CA group. Early-onset sepsis (EOS) occurred in 23 neonates (12.2%), with a significantly higher incidence in the CA group compared to the non-CA group (21% vs. 8%, *p* = 0.014). Late-onset sepsis (LOS) developed in 66 neonates (34.9%), but no significant difference in incidence was observed between the two groups (*p* = 0.402). Parsimonious logistic regression analysis identified maternal chorioamnionitis as an independent predictor of EOS (Odds Ratio 2.07, 95% CI 1.85–5.08; *p* = 0.009). **Conclusions**: Intrauterine infection and inflammation caused by chorioamnionitis are linked to an increased risk of early-onset sepsis in neonates born before 32 weeks’ gestation. However, chorioamnionitis does not appear to significantly influence the incidence of late-onset sepsis, which appears to be more closely associated with postnatal factors.

## 1. Introduction

Prematurity remains a significant global health concern, posing ongoing challenges for obstetricians, neonatologists, and other healthcare providers. Preterm labor is a clinical syndrome with diverse etiologies, leading to premature birth in approximately 70% of cases [1,2]. The underlying causes of prematurity are primarily grouped into two pathological categories: intrauterine infection and/or inflammation, and placental vascular disorders. Among these, intrauterine infection is strongly associated with chorioamnionitis (CA) and various maternal complications [3].

### 1.1. The Epidemiology of Prematurity

The reported incidence of CA ranges from 1% to 10% of all pregnancies and is inversely related to gestational age. It is implicated in up to 40–70% of preterm births [4,5,6]. CA can affect multiple fetal organs and is associated with increased maternal and neonatal morbidity and mortality [4,7]. In particular, it has been linked to spontaneous preterm labor, premature rupture of membranes (PROM), and adverse perinatal outcomes, especially in very low birth weight infants [1,8,9].

### 1.2. Chorioamnionitis and Triple I

The term “chorioamnionitis” alone does not adequately capture the severity, duration, or extent of the inflammatory or infectious process, complicating the prediction of maternal and neonatal outcomes [4,10]. To address this, a broader term—Triple I (intrauterine infection or inflammation or both)—has been introduced to better reflect the heterogeneous nature of these conditions and their clinical implications [4,10]. CA is further classified into clinical and histological types. Histological chorioamnionitis (HCA) is defined by an intrauterine inflammatory response characterized by diffuse neutrophilic infiltration of maternal and/or fetal tissues [11]. Beyond its role in triggering preterm birth, CA is associated with a 2- to 3.5-fold increased risk of adverse neonatal outcomes, including neonatal sepsis [12]. This correlation is likely mediated through immune system dysregulation and fetal immune programming during intrauterine life.

### 1.3. Neonatal Sepsis (EOS and LOS)

Neonatal sepsis remains a leading cause of neonatal morbidity and mortality worldwide and is a priority focus of the World Health Organization (WHO) [13,14]. The incidence of neonatal sepsis varies dramatically depending on a country’s socioeconomic context, ranging from 1 to 4 to as high as 49–170 cases per 1000 live births, with mortality rates exceeding 24% in some regions [13]. Neonatal sepsis is a clinical syndrome characterized by nonspecific signs and symptoms resulting from pathogen invasion. Diagnosis requires both clinical assessment and laboratory confirmation [15,16]. Early-onset sepsis (EOS), defined as sepsis occurring within the first 72 h of life, is particularly concerning in preterm infants. CA is a well-established antecedent of EOS, most often due to vertical transmission of pathogens and the fetal inflammatory response syndrome [4,17,18]. However, not all neonates born to mothers with CA develop EOS, and identifying those at highest risk remains a major clinical challenge. In contrast, late-onset sepsis (LOS)—defined as sepsis occurring after 72 h of life is more frequently associated with postnatal risk factors such as prolonged hospitalization, invasive procedures, and nosocomial infections. Its association with antenatal infections like CA is less clearly defined [19].

### 1.4. Study Rationale and Objectives

Although several studies have explored the relationship between CA and EOS, many suffer from methodological limitations, including heterogeneous study populations, outdated definitions, and lack of adjustment for key confounding factors such as gestational age, birth weight, and use of central venous catheters. Moreover, data specifically focusing on very preterm neonates (<32 weeks gestation)—a population uniquely susceptible to both intrauterine infection and postnatal complications due to immunologic immaturity and prolonged intensive care exposure—remain limited [20]. While the association between CA and EOS is established, few contemporary studies focus specifically on the <32-week population using current standardized criteria while adjusting for modern NICU interventions like UVC/UAC placement. Given the updated diagnostic criteria for CA outlined by the American College of Obstetricians and Gynecologists (ACOG) and the Vermont Oxford Network, there is a clear need for contemporary studies to reassess previous findings using standardized definitions and modern clinical practices [21]. The operational definitions for early-onset sepsis (EOS) and late-onset sepsis (LOS) used in our analysis were the standardized VON criteria in effect during the 2020–2022 study period [22]. In alignment with the updated literature, the current research focused on the clear definition of chorioamnionitis and sepsis before conducting the investigation [22]. The primary aim of the present study was to evaluate the association between chorioamnionitis and the incidence of possible and culture-proven early-onset and late-onset neonatal sepsis in preterm neonates born at <32 weeks’ gestation.

While the association between maternal chorioamnionitis and early-onset neonatal sepsis has been reported in previous studies and meta-analyses, data focusing specifically on very preterm neonates born at <32 weeks’ gestation remains comparatively limited. Given the unique immunological vulnerability and clinical course of this population, further evaluation of EOS risk in this subgroup is warranted. Therefore, the present study aimed to investigate the association between microbiologically confirmed maternal chorioamnionitis and early-onset sepsis specifically in very preterm infants. Under this framework, the secondary purpose was to investigate further the relationship between maternal CA and early-onset sepsis, taking into account relevant maternal and neonatal factors.

## 2. Materials and Methods

This research constituted a retrospective observational 3-year study in a single university perinatal center, focusing on the investigation of neonates born at <32 weeks’ gestational age (GA). Electronic medical records of all newborns and the respective maternal medical files were reviewed, and specific inclusion criteria were applied. These were the following: (a) gestational age < 32 weeks, (b) examination for chorioamnionitis, (c) complete maternal medical files. A power analysis revealed that, based on the calculated EOS proportion in our cohort, a total sample size of 177 neonates would be sufficient to detect a difference between the two groups, with a power of 0.8 and a type-I error of 0.05.

Chorioamnionitis was defined by the presence of either microbiological evidence, based on positive amniotic fluid results obtained before or at delivery (including Gram stain, glucose levels, or positive cultures), or histological evidence from placental examination demonstrating inflammation, such as acute neutrophilic infiltration of maternal and/or fetal tissues [11,21]. Infants without available microbiological results or placental biopsy examination, as well as incomplete neonatal or maternal medical reports, were excluded from the study. Medical records were reviewed, and infants as well as their mothers were divided into two groups: those with confirmed chorioamnionitis (CA group), and those without evidence of CA (non-CA group). The clinical definition of sepsis was according to Vermont Oxford Network criteria [22], as the presence of one or more signs of systemic infection (e.g., apnea, temperature instability, feeding intolerance, respiratory distress, hemodynamic instability), and treatment with at least 5 days of intravenous antibiotics following blood culture collection—regardless of culture result. The confirmation of sepsis was defined by microbial growth in blood or another sterile site (e.g., CSF, urine, peritoneal or pleural fluid) [20]. Early-onset sepsis (EOS) typically occurs within the first 72 h of life, while late-onset sepsis (LOS) presents after the first 72 h of life [22]. We define as premature rupture of membranes (PROM) the rupture lasting ≥18 h. To minimize diagnostic bias associated with the VON sepsis definition (which includes 5 days of antibiotic therapy), clinical management was dictated by a standardized institutional protocol. The decision to continue antibiotics beyond 48–72 h was based on the persistence of clinical symptoms and serial inflammatory markers (CRP) rather than the presence of chorioamnionitis or PROM alone.

The operational definitions for early-onset sepsis (EOS) and late-onset sepsis (LOS) used in our analysis were the standardized VON criteria in effect during the 2020–2022 study period [22]. The updates were referenced to demonstrate that our findings remain aligned with the most current international reporting standards; however, the actual data collection and patient classification were performed using the criteria active at the time of the study (VON Release 23.2. Published 2019) [22]. All the reports were obtained from the computerized documentation system of the hospital and included maternal and neonatal data. In specific, the maternal data contained information for maternal age, the parity, the mode of delivery, the incidence of premature rupture of membranes (PROM), the need for antenatal corticosteroid or antibiotic administration, the laboratory markers of infection (C-reactive protein, white blood cell count) and the placental histopathology.

The neonatal records concerned the gestational age and sex, the birth weight and its percentiles, the Apgar scores (1st and 5th minute), resuscitation measures at birth (non-invasive and invasive), the incidence of patent ductus arteriosus (PDA), the type and duration of central vascular catheter placement [umbilical venous catheter (UVC), umbilical arterial catheter (UAC), peripherally inserted central catheter (PICC)] and the complications associated with central lines (e.g., CLABSI, thrombosis, skin erosion). Prior to data collection, approval was obtained from the Scientific Board of Hospital. Furthermore, all data was used exclusively for the purposes of the present research, and full anonymity of participants was ensured throughout the study.

### Statistical Analysis

Descriptive statistics were calculated for maternal and neonatal characteristics. The normality of the distributions of continuous variables was assessed with the Shapiro–Wilk’s normality test. Categorical variables were expressed as counts (*n*) and percentages (%). Continuous variables were expressed as mean and standard deviation if normally distributed and as median and interquartile range (IQR) if not normally distributed. Categorical variables were compared between groups using chi-square or Fisher’s exact test where appropriate. Continuous variables were compared using Mann–Whitney U test for non-parametric continuous variables, and independent sample *t*-Test for parametric variables. A parsimonious logistic regression model was conducted to examine the association of EOS with maternal CA, adjusted for gestational age, sex, birth weight, antenatal steroid administration, intubation, UAC and UVC placement, using backward stepwise selection of variables with *p* < 0.05. Goodness-of-fit was conducted with the Cox & Snell R Square, the Nagelkerke R Square, and the Hosmer and Lemeshow test, as provided in Supplementary Tables S1 and S2. All performed tests were two-sided and a *p*-value less than 0.05 was considered statistically significant (alpha 0.05). The data were analyzed using SPSS Statistics Version 26.0 (IBM, Chicago, IL, USA).

## 3. Results

**Figure 1:**
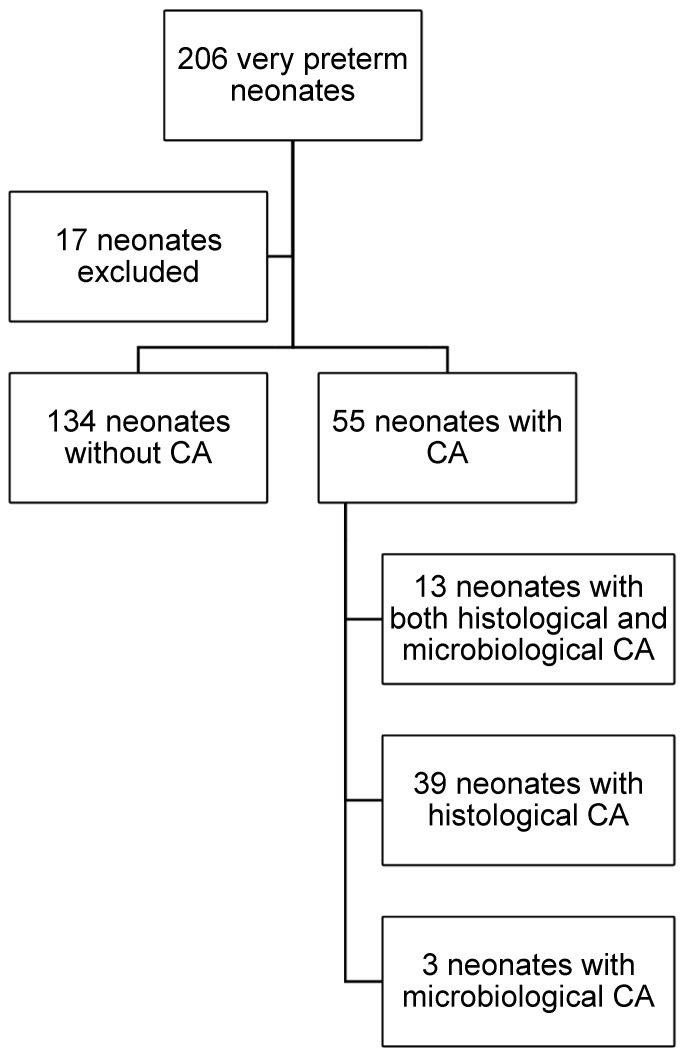
Flowchart of the study population.

During the study period a total of 206 very preterm neonates with gestational age < 32 weeks were born in our institution. After excluding 17 neonates due to missing placental histological examination or incomplete medical records, 189 neonates were included in the study, and their medical files were carefully investigated (Figure 1).Baseline maternal characteristics are presented in Table 1. Maternal age and mode of delivery did not differ significantly between the CA and non-CA groups. Similarly, the proportion of primiparous women were comparable across groups. In contrast, the prolonged rupture of membranes was significantly more frequent among mothers with CA (21%) than among those without CA (10%), *p* = 0.034. Antenatal corticosteroid administration was significantly less common in the CA group compared with the non-CA group, *p* = 0.017, while antenatal antibiotic use showed an increased trend in the CA group, although this did not reach statistical significance, *p* = 0.054. Markers of maternal inflammation were significantly elevated in the CA group. Maternal white blood cell counts, and maximum maternal C-reactive protein levels were higher in mothers with CA compared with those without CA, with significant difference *p* < 0.001 and *p* = 0.001, respectively. Among the 55 cases of CA, the majority were diagnosed histologically, with smaller proportions identified microbiologically or by both methods. The mean GA of the cohort was 29.2 ± 2.1 weeks, and the mean birth weight was 1286 ± 405 g. Of the 189 enrolled neonates, 16 (8.5%) died within the first three days of life and were included in the EOS analysis. Approximately half of them were male (45.5%) and the 54.5% of them were female. Among the 189 neonates, 55 (29.1%) were born to mothers with confirmed chorioamnionitis (CA group), and 134 (70.9%) were born without evidence of CA (non-CA group). Comparative data on neonatal characteristics between the CA and non-CA groups are shown in Table 2. Neonates in the CA group had significantly lower GA (*p* < 0.001) and significantly lower birth weight (*p* < 0.016). Intubation at birth was more frequent in neonates with CA (*p* = 0.040), as well as patent ductus arteriosus (PDA) (*p* = 0.039).

### 3.1. Infection and Mortality Outcomes

Regarding neonatal infections, 23 neonates (12.2%) developed early-onset sepsis (EOS), while 66 neonates (34.9%) developed late-onset sepsis (LOS). EOS occurred significantly more frequently among neonates exposed to chorioamnionitis (21%) compared with those without CA exposure (8%), *p* = 0.014. In contrast, no significant difference was observed in the incidence of LOS between the two groups (*p* = 0.402). Regarding neonatal mortality, there was a trend toward higher mortality in the CA group (14%) than in the non-CA group (6%), without a statistically significant difference (*p* = 0.081).

### 3.2. Risk Factors for Early-Onset Sepsis

The risk factors associated with the development of early-onset sepsis (EOS), as investigated by parsimonious logistic regression analysis, are presented in Table 3. Maternal chorioamnionitis (CA) was significantly associated with an increased risk of EOS (OR 2.07, 95% CI 1.85–5.08; *p* = 0.009). Of the 23 EOS cases identified, three (13%) were culture-proven, all of which occurred within the CA group. The identified pathogens included Acinetobacter baumannii, Streptococcus agalactiae, and Staphylococcus epidermidis. The remaining 20 cases met the clinical and laboratory criteria for EOS despite sterile blood cultures. Even though birth weight and UVC placement are often thought of as risk factors, data showed they were not statistically significant (*p* > 0.05) when adjusted for other variables in this specific model; each additional week of gestation conferred a reduced risk (OR 0.79, 95% CI 0.73–0.86; *p* = 0.001). Furthermore, male sex was associated with a higher likelihood of EOS (OR 3.59, 95% CI 1.23–10.40; *p* = 0.019). Conversely, birth weight was not statistically significant (OR 1.00, 95% CI 0.98–1.02; *p* > 0.05). Regarding markers of illness severity and invasive support, umbilical arterial catheter (UAC) placement demonstrated the strongest association as a significant predictor of EOS (OR 5.41, 95% CI 2.20–13.31; *p* = 0.003). However, umbilical venous catheter (UVC) placement (OR 1.05, 95% CI 0.36–3.09; *p* = 0.920) and intubation (OR 1.18, 95% CI 0.34–4.06; *p* = 0.792) were not significantly associated with the development of EOS.

## 4. Discussion

The current retrospective cohort study examined the association between maternal chorioamnionitis (CA) and the risk of early- and late-onset neonatal sepsis among very preterm infants born before 32 weeks of gestation. It should also be acknowledged that a substantial proportion of EOS cases in this cohort were classified as clinical EOS according to VON criteria rather than culture-proven infection. This pattern is consistent with the broader neonatal sepsis literature, where culture-negative but clinically treated EOS frequently exceeds microbiologically confirmed cases. Several factors contribute to this phenomenon, including the limited blood culture volumes obtained in neonates, low-level bacteremia, and the potential influence of early antibiotic exposure. Therefore, the use of combined culture-proven and guideline-defined clinical EOS criteria reflects current epidemiological practice and allows a more comprehensive assessment of neonatal infection in the context of confirmed maternal chorioamnionitis exposure. Accordingly, the present analysis was not designed to support pathogen-specific causal inference, but rather to evaluate the overall risk and occurrence of EOS among neonates exposed to microbiologically confirmed maternal infection, using outcome definitions consistent with established neonatal surveillance frameworks. Our findings indicate that maternal CA is significantly associated with an increased risk of early-onset sepsis (EOS), but not late-onset sepsis (LOS), in this vulnerable population. Notably, after adjustment for potential maternal and neonatal confounders in parsimonious logistic regression analysis, CA remained an independent predictor of EOS, but not for the development of LOS. Collectively, these findings underscore the pivotal role of intrauterine infection and inflammation in the pathogenesis of early neonatal infections, while suggesting a more limited contribution to late-onset sepsis [23]. Within this context, the findings of our study are in line with the etiologically based functional classification proposed by José Villar et al., which factors and frames preterm birth as a heterogeneous, multifactorial syndrome with distinct underlying pathways. According to this model, intrauterine infection and inflammation represent a major pathogenic mechanism, directly linked to adverse early neonatal outcomes, including early-onset sepsis [2]. Our findings are consistent with prior studies. Beck et al. reported an increased risk of EOS in neonates exposed to any form of CA, which varies from three-fold to six-fold [24]. On the same wavelength, a systematic review and meta-analysis by Villamor-Martinez et al., including 107 studies, similarly confirmed a strong association between CA and EOS [3]. Several pathophysiological mechanisms have been proposed to explain this association.

The immunomodulatory effects of intrauterine inflammation on the neonate are likely to contribute, altering both immune development and susceptibility to infection [3]. Additionally, vertical transmission of pathogens through placenta represents a biologically plausible pathway for early-onset sepsis, particularly in the setting of prolonged rupture of membranes (PROM) [3,24], a finding which was significantly more common in the CA group in our cohort (*p* = 0.034). In contrast, LOS is more commonly attributed to postnatal factors such as prolonged hospitalization, invasive procedures, and nosocomial exposures. The absence of a significant association between CA and LOS in our study supports this statement and highlights the differing pathophysiological mechanisms between EOS and LOS [19].

The two-fold increased odds of EOS in neonates born to mothers with CA underscore the need for vigilant clinical surveillance and timely empiric antibiotic administration in this population. Perinatal management strategies for very preterm infants should include prompt recognition of CA—whether clinical, histological, or microbiological—given its significant implications for neonatal outcomes [4].

A critical distinction must be made between neonatal sepsis resulting from direct bacterial invasion and the systemic inflammatory state induced by intrauterine cytokine exposure. Our findings demonstrate a significant association between chorioamnionitis and clinical EOS, despite a low yield of positive blood cultures. This clinical phenotype likely reflects the Fetal Inflammatory Response Syndrome (FIRS), a condition where the fetus is exposed to a ‘cytokine storm’ (specifically IL-1, IL-6, and TNF-α) within the gestational sac. Even in the absence of viable bacteria in the neonatal bloodstream, these pro-inflammatory mediators can trigger systemic symptoms—such as respiratory distress, temperature instability, and hematological abnormalities—that meet the clinical criteria for sepsis. Therefore, the association observed in this study may represent the biological continuum of intrauterine inflammation rather than solely vertical bacterial transmission [25]. This underscores the importance of monitoring infants exposed to chorioamnionitis for systemic inflammatory consequences, even when microbiologic confirmation is lacking. Another interesting finding is that neonates in the CA group also had higher rates of respiratory and cardiovascular complications, such as increased intubation in the delivery room (*p* = 0.040) and a higher incidence of patent ductus arteriosus (*p* = 0.039). These outcomes likely reflect the lower gestational age and birth weight observed in this group. The observed trend toward higher mortality in the CA group (14% vs. 6%, *p* = 0.081), although not statistically significant, may further indicate additionally the broader clinical burden associated with intrauterine infection.

An important question arises as to why it remains clinically and scientifically relevant to examine the association between early-onset sepsis (EOS) and chorioamnionitis (CA), despite this relationship being well established in the literature. While the link between CA and EOS has been previously documented, our study adds meaningful insight in several key respects. The high proportion of culture-negative EOS (87%) observed in this study is a recognized challenge in neonatal medicine. The low yield of blood cultures in this cohort may be attributed to several factors, including the low volume of blood typically drawn from preterm neonates and the high rate of maternal intrapartum antibiotic exposure in the CA group, which can suppress bacterial growth. Furthermore, the clinical presentation of EOS in infants exposed to CA may reflect the systemic effects of pro-inflammatory cytokines (Fetal Inflammatory Response Syndrome) rather than viable bacteremia alone. Consequently, while our data strongly links CA to the clinical syndrome of EOS, we acknowledge that the small number of culture-proven cases limits our ability to draw definitive conclusions regarding specific pathogen-driven causality.

We focused exclusively on very preterm neonates born before 32 weeks of gestational age, a population that is uniquely vulnerable to intrauterine infection, systemic inflammation, and adverse infectious outcomes. Many prior studies included heterogeneous cohorts encompassing term and preterm infants, thereby limiting the applicability of their findings to this particularly high-risk group. Moreover, we employed contemporary diagnostic criteria for both CA and EOS, consistent with the updates from the American College of Obstetricians and Gynecologists (ACOG) and the Vermont Oxford Network [22]. In contrast, earlier investigations often relied on outdated or inconsistent definitions, which may reduce their relevance to current clinical practice. The operational definitions for early-onset sepsis (EOS) and late-onset sepsis (LOS) used in our analysis were the standardized VON criteria in effect during the 2020–2022 study period [22].

Finally, although the single-center design may limit generalizability, it allows for de- tailed insight of local clinical practices, patient demographics, and outcomes. Such data are valuable for institutional benchmarking, informing local clinical protocols, and con- tributing high-quality evidence to future multicenter studies and meta-analyses.

Our findings clearly distinguish the impact of CA on EOS—but not LOS—reinforcing the concept that LOS is more strongly influenced by postnatal care, such as central line use and NICU environment, rather than intrauterine exposures. Although not statistically significant, the trend toward increased early mortality in neonates with CA may warrant further investigation, particularly in relation to severity and duration of intrauterine infection. Mortality rate did not reach statistical significance, but the 2.3-fold difference in mortality suggests a clinical trend that may be significant in a larger multicenter cohort. Even when findings are confirmatory, high-quality observational data are essential for future systematic reviews and meta-analyses that inform clinical guidelines and policy development. In the parsimonious logistic regression analysis, UAC placement had a high Odds Ratio of 5.41 for EOS. We believe UAC placement is not a cause of EOS but a marker of illness severity. Since EOS occurs within 72 h, a catheter placed at birth is often a response to a neonate who already appears ill (the “illness-marker” hypothesis).

Our findings are further supported by our previous research, which demonstrated that while clinical chorioamnionitis is a significant risk factor, histological chorioamnionitis (HCA) provides a more definitive association with the development of EOS [26]. In that study, we observed that clinical suspicion of chorioamnionitis often does not correlate perfectly with histological findings, yet the presence of histological inflammation—particularly when associated with a fetal inflammatory response syndrome (FIRS)—is a potent predictor of early neonatal infection. By focusing on a more immature cohort in the current study (<32 weeks), we reinforce the necessity of using standardized ‘Triple I’ terminology (Intrauterine Inflammation or Infection or both) to better categorize these high-risk neonates and guide clinical management. Although the association between chorioamnionitis (CA) and early-onset sepsis (EOS) is well established, several clinically relevant questions remain unanswered. Specifically, it is unclear which subtypes of CA—clinical, histological, or microbiological—are associated with the highest risk of EOS, and to what extent current antenatal interventions, such as corticosteroid or antibiotic therapy, can modify this risk [27,28]. The difference in antenatal antibiotic use between groups (*p* = 0.054) is nearly significant, noting that the higher (though non-significant) use of antibiotics in the CA group might have contributed to the low rate of culture-proven EOS (only three cases) by partially treating the infant in utero. Similarly, the early empirical neonatal antibiotic therapy may have reduced blood culture sensitivity and contributed to a high proportion of culture-negative EOS. Furthermore, the long-term consequences of in utero exposure to CA on immune system maturation, neonatal microbiome development, and neurodevelopmental outcomes remain incompletely understood [4,29,30,31]. Future research should address these questions through prospective, multicenter studies with standardized definitions and larger sample sizes.

A significant finding in our demographic data was the lower rate of antenatal steroid (ANS) administration in mothers with chorioamnionitis. This discrepancy is frequently observed in clinical practice, as the presence of infection often mandates immediate delivery, leaving insufficient time for a 48 h steroid course. Since ANS are known to modulate the neonatal inflammatory response and improve overall stability, their relative absence in the CA group may have contributed to the higher severity of illness observed. However, our parsimonious logistic regression analysis demonstrates that even when controlling ANS exposure, CA did not exert a significant independent effect on the risk of early-onset sepsis. This suggests that the pro-inflammatory milieu of CA carries a risk that is not entirely mitigated by the administration of corticosteroids.

The practical significance of our findings lies in the clinical stratification of high-risk preterm neonates. By identifying maternal chorioamnionitis as a significant independent predictor of early-onset sepsis (OR 2.07), our study reinforces the necessity for a low threshold for initiating empiric antibiotic therapy in infants born at <32 weeks’ gestation exposed to intrauterine inflammation. Furthermore, the strong association with UAC placement (OR 5.41) serves as a vital clinical ‘red flag’; clinicians should recognize that the need for arterial access in the first hours of life is often the first sign of a neonate already in the early stages of a systemic inflammatory response. Conversely, the lack of association between CA and LOS suggests that prevention of late infections should focus primarily on postnatal stewardship, such as hand hygiene and central line maintenance, rather than prenatal history.

Finally, several limitations of the present study must be acknowledged. The principal limitation is its retrospective design, which may introduce selection or information bias. The single-center setting may affect generalizability and may not represent the epidemiology of other areas. Additionally, the exclusion of 17 neonates due to missing placental pathology or incomplete records could possibly introduce selection bias. Likewise, due to the retrospective nature of this study, certain maternal variables such as Body Mass Index (BMI) and specific genital tract microbiological swabs were not consistently documented for all participants and thus could not be included in the final parsimonious logistic regression analysis. Furthermore, the relatively small number of EOS cases limited the statistical power for subgroup analysis (e.g., comparing histological vs. microbiological CA).

This reflects the single-center nature of the cohort and the relatively low incidence of EOS, even among neonates exposed to maternal infection. Therefore, the multivariable analysis should be interpreted as exploratory, and larger multicenter studies are warranted to confirm these findings. A limitation of this study is the potential for treatment-driven bias inherent in the VON definition of neonatal sepsis. Because this definition includes a duration of ≥5 days of antibiotic treatment, it is possible that infants known to be high-risk due to chorioamnionitis exposure were treated more conservatively. However, the significantly higher rate of culture-proven cases and the severity of clinical symptoms in the CA group suggest that the observed association reflects a true biological increase in infection risk rather than solely a result of prolonged prophylactic treatment. Finally, we did not stratify CA according to staging or grading criteria, which may be important for understanding the relationship between severity of inflammation and neonatal outcomes. Furthermore, the lack of power to distinguish between cytokine-mediated vs. culture-positive inflammation is a limitation of the current retrospective design.

On the other hand, our study has specific strengths to outweigh the abovementioned limitations. These include the use of rigorous histological and microbiological definitions of chorioamnionitis, the adherence to current diagnostic guidelines from the American College of Obstetricians and Gynecologists (ACOG) and the Vermont Oxford Network (VON) and the careful selection of the sample. In addition, there was the application of parsimonious logistic regression analysis to adjust for key confounding variables, including gestational age, birth weight, and the need for invasive interventions.

## 5. Conclusions

In conclusion, maternal chorioamnionitis is significantly associated with an increased risk of early-onset neonatal sepsis among very preterm infants, even after adjustment for key perinatal and neonatal confounders. Our findings reinforce the need for prompt identification and management of intrauterine infection and underscore the importance of risk stratification in guiding early empiric antibiotic use and neonatal monitoring. Future prospective studies are needed to further characterize subtypes of CA, explore the mechanisms of infection transmission and immune modulation, and evaluate the impact of interventions aimed at mitigating the risk of neonatal sepsis.

## Figures and Tables

**Table 1:** Baseline maternal characteristics with and without CA.

	CA (n = 55)	Non-CA (n = 134)	*p*
Maternal age, years	34.2 ± 7.0	34.3 ± 6.7	0.945
Mode of delivery, vaginal delivery	5 (9%)	7 (5%)	0.336
Prolonged rupture of membranes	12 (21%)	13 (10%)	0.034
Primiparity	41 (74%)	87 (65%)	0.714
Antenatal administration of steroids	21 (38%)	77 (57%)	0.017
Antenatal administration of antibiotics	30 (54%)	52 (38%)	0.054
Maternal WBC count	15,450 (12,800–18,600)	12,600 (10,100–14,800)	<0.001
Maternal CRP max	2.14 (0.57–4.85)	0.74 (0.40–1.35)	0.001
CA	55	-	<0.001
Microbiological	3 (2%)		
Histological	39 (20%)		
Both	13 (7%)		

Continuous variables are presented as mean ± standard deviation or median (IQR); categorical variables are presented as numbers (%). χ^2^-tests and Fisher exact tests were performed for categorical variables; Mann–Whitney and *t*-Tests were performed for continuous variables. CA, chorioamnionitis; WBC, white blood cells; CRP, C-reactive protein.

**Table 2:** Baseline neonatal characteristics with and without CA.

	CA (n = 55)	Non-CA (n = 134)	*p* †
Gestational age, weeks	27.9 ± 1.8	29.7 ± 2.4	<0.001
Extremely preterm neonates	23 (42%)	17 (12%)	<0.001
Sex, male	25 (45%)	61 (45%)	1.000
Birth weight, g	1166 ± 448	1335 ± 377	0.016
Extreme low birth weight	23 (41%)	26 (19%)	0.002
Deviant birth weight			0.967
Small for gestational age	5 (9%)	13 (10%)	
Appropriate for gestational age	48 (87%)	117 (87%)	
Large for gestational age	2 (4%)	4 (3%)	
Apgar score at 1st min	7 (6–7)	7 (6–8)	0.111
Apgar score at 5th min	8 (8–8)	8 (8–8)	0.382
Resuscitation	47 (85%)	108 (80%)	0.534
Intubation	16 (29%)	20 (15%)	0.040
Patent ductus arteriosus	15 (27%)	19 (14%)	0.039
Respiratory distress syndrome	33 (60%)	85 (63%)	0.741
Early-onset sepsis	12 (21%)	11 (8%)	0.014
Blood-positive early-onset sepsis	3 (13%)	-	0.027
Late onset sepsis	22 (40%)	44 (32%)	0.402
Blood-positive late-onset sepsis	13 (39%)	26 (39%)	1.000
UAC	20 (36%)	41 (30%)	0.494
UAC duration (days)	0 (0–7)	0 (0–3)	0.324
UAC complications	3 (5%)	2 (1%)	0.149
UVC	27 (49%)	53 (39%)	0.258
UVC duration (days)	9 (7–10)	8 (6–10)	0.470
UVC complications	6 (11%)	6 (4%)	0.110
PICC	28 (50%)	65 (48%)	0.873
PICC duration (days)	12 (8–16)	10 (7–16)	0.205
PICC complications	2 (3%)	5 (3%)	1.000
Duration of stay, days	48 (33–82)	38 (29–55)	0.024
Mortality	8 (14%)	8 (6%)	0.081

Continuous variables are presented as mean ± standard deviation or median (IQR); categorical variables are presented as numbers (%). χ^2^-tests and Fisher exact tests were performed for categorical variables; Mann–Whitney and *t*-Tests were performed for continuous variables. CA, chorioamnionitis; UAC, umbilical arterial catheter; UVC, umbilical venous catheter; PICC, percutaneous inserted central catheter. †: represents *p* < 0.05 considered statistically significant

**Table 3:** *Parsimonious* logistic regression model using backward stepwise selection of variables with *p* < 0.05, examining the association of EOS with maternal CA, adjusted for gestational age, sex, birth weight, antenatal steroid administration, intubation, UAC and UVC placement.

	*p*	Odds Ratio	95% CI
CA	0.009	2.07	1.85–5.08
Sex	0.019	3.59	1.23–10.40
Gestational age, weeks	0.001	0.79	0.73–0.86
Birth weight, grams	0.741	1.00	0.98–1.02
Antenatal steroids administration	0.415	1.51	0.56–4.08
Intubation	0.792	1.18	0.34–4.06
UAC	0.003	5.41	2.20–13.31
UVC	0.920	1.05	0.36–3.09

CA, chorioamnionitis; CI, confidence intervals; UAC, umbilical arterial catheter; UVC, umbilical venous catheter.

## Data Availability

All authors of the published article could share their research data.

## Supplementary Materials

The following supporting information can be downloaded at: https://www.mdpi.com/article/10.3390/diagnostics16081125/s1, Table S1: Model summary of the parsimonious logistic regression model; Table S2: Hosmer and Lemeshow Test of the parsimonious logistic regression model.

## Abbreviations

The following abbreviations are used in this manuscript:

CA	Chorioamnionitis
EOS	early-onset sepsis
LOS	late-onset sepsis
NICUs	neonatal intensive care units
ACOG	American College of Obstetricians and Gynecologists
VON	Vermont Oxford Network
PROM	premature rupture of membranes
HCA	Histological chorioamnionitis
CSF	Cerebrospinal Fluid
CLABSI	Central Line-Associated Bloodstream Infection
