# Supplementary material for: Association of Chorioamnionitis with Early and Late Neonatal Sepsis in Preterm Infants with Gestational Age < 32 Weeks
Source: Diagnostics (Basel). 2026 Apr 9;16(8):1125. doi: 10.3390/diagnostics16081125 (PMC13115544; doi:10.3390/diagnostics16081125)
Supplement: Supplementary file 1 [file diagnostics-16-01125-s001.zip › diagnostics-4204225-supplementary.pdf]

Supplemental Tables

Supplemental Table S1. Model summary of the parsimonious logistic regression model.

Model Summary			
Step	-2 Log likelihood	Cox & Snell R Square	Nagelkerke R Square
1	105.336[a]	0.562	0.750
2	105.346[a]	0.562	0.750
3	105.415[a]	0.562	0.749
4	105.524[a]	0.562	0.749
5	106.197[a]	0.560	0.747
6	108.748[a]	0.554	0.739

a. Estimation terminated at iteration number 6 because parameter estimates changed by less than 0.001.

Supplemental Table S2. Hosmer and Lemeshow test of the parsimonious logistic regression model.

Hosmer and Lemeshow Test			
Step	Chi-square	df	Significance
1	2.749	8	0.949
2	2.738	8	0.950
3	1.363	8	0.995
4	3.188	8	0.922
5	6.424	8	0.600
6	3.279	8	0.916
